# Glial and neuronal expression of the Inward Rectifying Potassium Channel Kir7.1 in the adult mouse brain

**DOI:** 10.1111/joa.13048

**Published:** 2019-07-15

**Authors:** Maria Papanikolaou, Anthony Lewis, Arthur M. Butt

**Affiliations:** ^1^ Institute of Biomedical and Biomolecular Sciences School of Pharmacy and Biomedical Science University of Portsmouth Portsmouth UK

**Keywords:** astrocytes, immunohistochemistry, Kir7.1, neurones, oligodendrocytes

## Abstract

Inward Rectifying Potassium channels (Kir) are a large family of ion channels that play key roles in ion homeostasis and neuronal excitability. The most recently described Kir subtype is Kir7.1, which is known as a K^+^ transporting subtype. Earlier studies localised Kir7.1 to subpopulations of neurones in the brain. However, the pattern of Kir7.1 expression across the brain has not previously been examined. Here, we have determined neuronal and glial expression of Kir7.1 in the adult mouse brain, using immunohistochemistry and transgenic mouse lines expressing reporters specific for astrocytes [glial fibrillary acidic protein‐enhanced green fluorescent protein (GFAP‐EGFP], myelinating oligodendrocytes (PLP‐DsRed), oligodendrocyte progenitor cells (OPC,* Pdgfra‐cre* *ER*^*T*^ ^*2*^/*Rosa26‐YFP* double‐transgenic mice) and all oligodendrocyte lineage cells (SOX10‐EGFP). The results demonstrate significant neuronal Kir7.1 immunostaining in the cortex, hippocampus, cerebellum and pons, as well as the striatum and hypothalamus. In addition, astrocytes are shown to be immunopositive for Kir7.1 throughout grey and white matter, with dense immunostaining on cell somata, primary processes and perivascular end‐feet. Immunostaining for Kir7.1 was observed in oligodendrocytes, myelin and OPCs throughout the brain, although immunostaining was heterogeneous. Neuronal and glial expression of Kir7.1 is confirmed using neurone‐glial cortical cultures and optic nerve glial cultures. Notably, Kir7.1 have been shown to regulate the excitability of thalamic neurones and our results indicate this may be a widespread function of Kir7.1 in neurones throughout the brain. Moreover, based on the function of Kir7.1 in multiple transporting epithelia, Kir7.1 are likely to play an equivalent role in the primary glial function of K^+^ homeostasis. Our results indicate Kir7.1 are far more pervasive in the brain than previously recognised and have potential importance in regulating neuronal and glial function.

## Introduction

There are seven subfamilies of Kir channels, Kir1–7, and each subfamily has multiple members that can form as homotetramers or heterotetramers (Lagrutta et al. 1996; Pessia et al. 1996; Rojas et al. 2007). Kir7.1 are the most recently described subtype and are known as K^+^ transporting Kir, together with Kir1.1, Kir4.1, Kir4.2 and Kir5.1. Expression of Kir7.1 has been demonstrated in multiple transporting epithelia, in the small intestine (Partiseti et al. 1998), choroid plexus (Doring et al. 1998) gastric parietal cells (Fujita et al. 2002; Malinowska et al. 2004), kidney (Ookata et al. 2000; Derst et al. 2001; Suzuki et al. 2003), thyroid follicular cells (Nakamura et al. 1999), and in the retinal pigment epithelium (RPE) (Kusaka et al. 2001; Shimura et al. 2001; Yang et al. 2003), where genetic mutations of Kir7.1 cause the eye pathologies Snowflake Vitreoretinal Degeneration (SVD) and Leber's congenital amaurosis (LCA) (Kumar & Pattnaik, 2014). In addition, Kir7.1 have been shown to control the excitability of uterine smooth muscle during pregnancy in mice (McCloskey et al. 2014). In the central nervous system (CNS), Kir7.1 have been identified in cerebellar Purkinje neurones and hippocampal pyramidal neurones (Krapivinsky et al. 1998), and more recently in hypothalamic neurones, where they play a role in regulating excitability (Ghamari‐Langroudi et al. 2015). Kir7.1 transcript and protein expression has been demonstrated using *in situ* hybridisation, immunohistochemistry, Western and Northern blot, microarray and reverse transcription polymerase chain reaction (rtPCR) in human, rat, mouse, rabbit, guinea pig, bovine, porcine and monkey (Doring et al. 1998; Ookata et al. 2000; Derst et al. 2001; Suzuki et al., 2003; Yasuda et al. 2003; Pondugula et al. 2006; Yang et al., 2003). However, the overall distribution of Kir7.1 in the CNS has not been characterised in any species and glial cells have been neglected in this context. Here, we demonstrate widespread Kir7.1 immunostaining in neurones and glia throughout the adult mouse brain.

## Methods

### Animals and preparation of tissue

Mice of either sex were used throughout in accordance with regulations issued by the Home Office of the United Kingdom under the Animals (Scientific Procedures) Act, 1986. Mouse strains used were: C57BL6/10 wild type; PLP‐DsRed transgenic reporter mice, in which expression of DsRed is driven by the oligodendrocyte‐specific proteolipid protein (*PLP*) gene promoter (Hirrlinger et al. 2005); GFEC‐EGFP transgenic reporter mice, in which expression of EGFP is driven by the astrocyte‐specific *GFAP* gene promoter (Nolte et al. 2001); SOX10‐EGFP transgenic reporter mice, in which expression of EGFP is driven by the oligodendrocyte lineage‐specific *SOX10* gene promoter (Matsuoka et al. 2005; Kessaris et al. 2006); and *Pdgfra*‐creERT^2^‐Rosa26‐YFP mice, in which Cre recombination and YFP expression is driven by the oligodendrocyte progenitor cell (OPC)‐specific gene Pdgfra, induced by administration of tamoxifen, as previously detailed (Rivers et al. 2008). Mice were humanely sacrificed by cervical dislocations under terminal anaesthetic, and brains, optic nerves and skeletal muscle were removed for subsequent analyses.

### Western blot

Whole cell protein content was extracted from adult mouse cerebellum and cortex; tissues were homogenised in RIPA buffer 1× complete mini protease inhibitor cocktail (Roche; Burgess Hill, UK) using a Bertin Minilys. Samples were centrifuged at 4 °C, at high speed (17 000 *g*) for 15 min and supernatant was transferred in clean Eppendorfs. Quantification of protein concentration was carried out using the bicinchoninic acid assay (Sigma) with a standard bovine serum albumin (BSA) concentration curve and UV spectrophotometer (POLAR star OPTIMA, BMG LabTech, Ortenberg, Germany). Samples were mixed with Laemmli buffer, heated at 70 °C for 10 min with β‐mercaptoethanol and 60 μg of protein per lane was loaded for 10% acrylamide sodium dodecyl sulphate polyacrylamide gel electrophoresis (SDS‐PAGE). Proteins were then electrophoretically transferred to a polyvinylidene difluoride membrane (Amersham), which was then incubated in blocking solution 5% w/v dried milk in TBS (150 mm NaCl, 10 mm Tris, pH7.4 with 1% w/v Tween 20). Incubation in rabbit anti‐Kir7.1 antibody at 1 : 200 (Alomone) was carried out overnight at 4 °C and, following washes, the secondary antibody horseradish peroxidase‐conjugated goat anti‐rabbit (Agilent; Santa Clara, CA, USA) was added at 1 : 5000 for 2 h at room temperature (RT); controls were preincubated with the competitive peptide from which the Kir7.1 antibody was raised. Extensive washing of the membranes in TBS with 1% w/v Tween 20 was performed after each incubation and immunocomplexes were detected using the Luminata Forte chemiluminescence HRP detection reagent (Millipore). Finally, mouse β‐actin (1 : 3000, Sigma) incubation for 30 min was used as a positive control, followed by 1 h incubation with HRP‐conjugated goat anti‐mouse (1 : 5000, Agilent).

### Glial cell cultures

Glial cell cultures were prepared from optic nerve explants, as previously described (Papanikolaou et al. 2017). Briefly, optic nerves from P7‐P12 mice were placed into dissecting medium consisting of high glucose Dulbecco's modified Eagle medium (DMEM) (Sigma‐D5671) containing 10% fetal calf serum (FCS; Life Technologies), l‐glutamine (Sigma) and 0.1% gentamycin (Life Technologies). Nerves were finely chopped with a scalpel blade and triturated with pipettes of decreasing diameter. The solution was then pipetted onto poly‐D‐lysine/laminin‐coated coverslips and replaced after 24 h with a low serum (0.5%) modified Bottenstein and Sato (B&S) medium (Bottenstein & Sato, 1979). Explant cultures were used for immunolabelling after 8–12 days *in vitro* (DIV).

### Primary cortical neuron cultures

Cortical neuronal cultures prepared from brains from P0–P2 wild‐type mice were collected in ice‐cold HBSS (Life Technologies) containing 1% Pen/Strep (Life Technologies), cortices were dissected under a dissection microscope (Leica MZ8) and the cortical pieces were placed in fresh ice‐cold HBSS. A gentle washing step in HBSS was repeated three times so that any remaining pieces of brain membranes were washed away. The cortical pieces were then incubated at 37 °C for 15–20 min in NB‐A medium (Life Technologies) supplemented with B27, Pen/Strep, L‐glutamine (Sigma) and 10% trypsin (Life Sciences). Following trypsinisation, the media was replaced with FCS (Life Technologies) and incubated at 37 °C for 5–10 min, which inactivated any remaining enzyme. The cortical pieces were then washed with NB‐A medium and were triturated with a fire‐polished glass pipette. Finally, the media were filtered through a 70‐μm nylon mesh filter (Miltenyi Biotec), cells were counted with a VI‐CELL™ Series Cell Viability Analyser (Beckman Coulter) and 10^6^ cells were seeded per 13 mm coverslip and grown as monolayers in an incubator at 37 °C under normoxic conditions (95% air/5% CO_2_). Media were changed every 3–4 days and neurones were used for immunolabelling after 10–14 DIV.

### Immunohistochemistry

Brains, optic nerves and skeletal muscle were immersion‐fixed in 4% paraformaldehyde (PFA), brains for 12–16 h and optic nerves and muscle for 1–2 h, and subsequently washed in phosphate‐buffered saline (PBS) prior to sectioning. For brains, 40‐ to 70‐μm‐thick sections were cut sagittaly using a vibratome (Leica). Optic nerves and muscle were cryoprotected in sucrose, prior to embedding in Cryo‐M‐Bed (Bright Instruments Company Ltd), and 20‐μm sections were cut with a Leica CM3050 S cryostat at −21 °C and transferred onto Polysine^®^ coated slides (Thermo‐Scientific). For cell cultures, coverslips were fixed for 20 min in 1% PFA and washed thoroughly in PBS and after this were treated the same as tissue sections. Antigen retrieval was performed by incubation in 0.1 m PB followed by 10 min in 0.2 mg/mL pepsin/0.2 m HCL at 37 °C for 10 min. Following washes in TBS, a blocking stage was performed using 20% normal goat serum (NGS) for 2 h at RT, followed by incubation overnight with primary antibodies in blocking solution containing Triton X (Sigma). Primary antibodies used were rabbit anti‐Kir7.1 at 1 : 300 (Alomone), rat anti‐ myelin basic protein (MBP) at 1 : 300 (Millipore), chicken anti‐GFAP at 1 : 500 (Chemicon), mouse β3‐Tubulin (Tuj1) at 1 : 300 (Millipore), guinea pig anti‐Kir4.1 at 1 : 300 (Alomone), rabbit anti‐Kir5.1 at 1 : 300 (Alomone), mouse anti‐PSD95 at 1 : 500 (Thermo Fisher Scientific) and mouse anti‐Na^+^/K^+^ ATPase α1 at 1 : 500 (Abcam). Samples were then washed three times in PBS and incubated with the appropriate secondary antibodies conjugated with Alexa Fluor 488, 568 and 649 (1 : 400, Life Technologies) or TRITC (1 : 100, Sigma) as well as with the DNA dye Hoechst Blue (Molecular Probes‐1 : 1000). Controls were performed by preabsorption with the appropriate blocking peptide or, where this was not available, by omission of the primary antibody. Following immunolabelling, coverslips/sections were mounted with Fluoromount‐G^®^ (Southern Biotech). Images were acquired using a Zeiss Axiovert LSM710 VIS405 confocal microscope, using multichannel sequential scanning, narrow bandwidths, and minimal laser power and gain to prevent cross‐talk between the channels and images were exported and assembled using volocity (Perkin Elmer) and gimp ‐ gnu image manipulation Program (free software for Windows), respectively. volocity 3D isosurface rendering applies indirect surface rendering, which identifies a surface around objects where all voxel intensity values are the same, and was used to better illustrate cell surface immunolabelling characteristics.

## Results

### Expression of Kir7.1 in the adult mouse cerebellum

**Figure 1:**
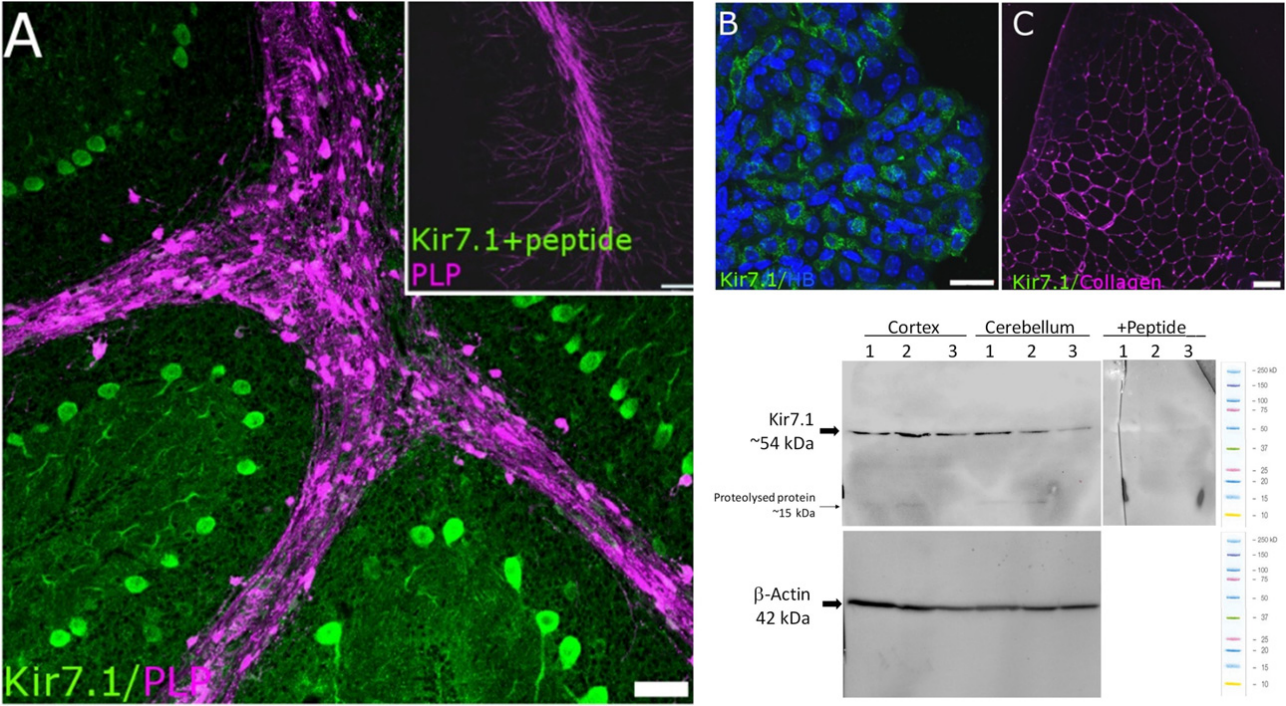
Validation of Kir7.1 immunostaining. (A‐C) To serve as positive controls, immunostaining for Kir7.1 is demonstrated in Purkinje neurones (A; adult PLP‐DsRed mouse cerebellum, in which white matter tracts appear magenta) and choroid plexus epithelium (B; counterstained with Hoechst Blue to visualise the cell nuclei). Immunostaining was absent following pre‐incubation in blocking peptide (A, Inset), and in adult mouse skeletal muscle, which does not express Kir7.1 (C; counterstained for collagen, which appears magenta). (D) Western blot analysis of protein lysates from mouse cerebellum (CBL) and cortex (CTX) confirmed robust Kir7.1 protein expression with a predicted band at 54 kDa. Positive bands were absent in the presence of the competitive peptide. In some samples, very dim bands were observed at approximately 15 kDa, which corresponds to protein proteolysed during sampling. Scale bars: (A,C) 50 μm; (B) 10 μm; (A, Inset) 100 μm.

**Figure 2:**
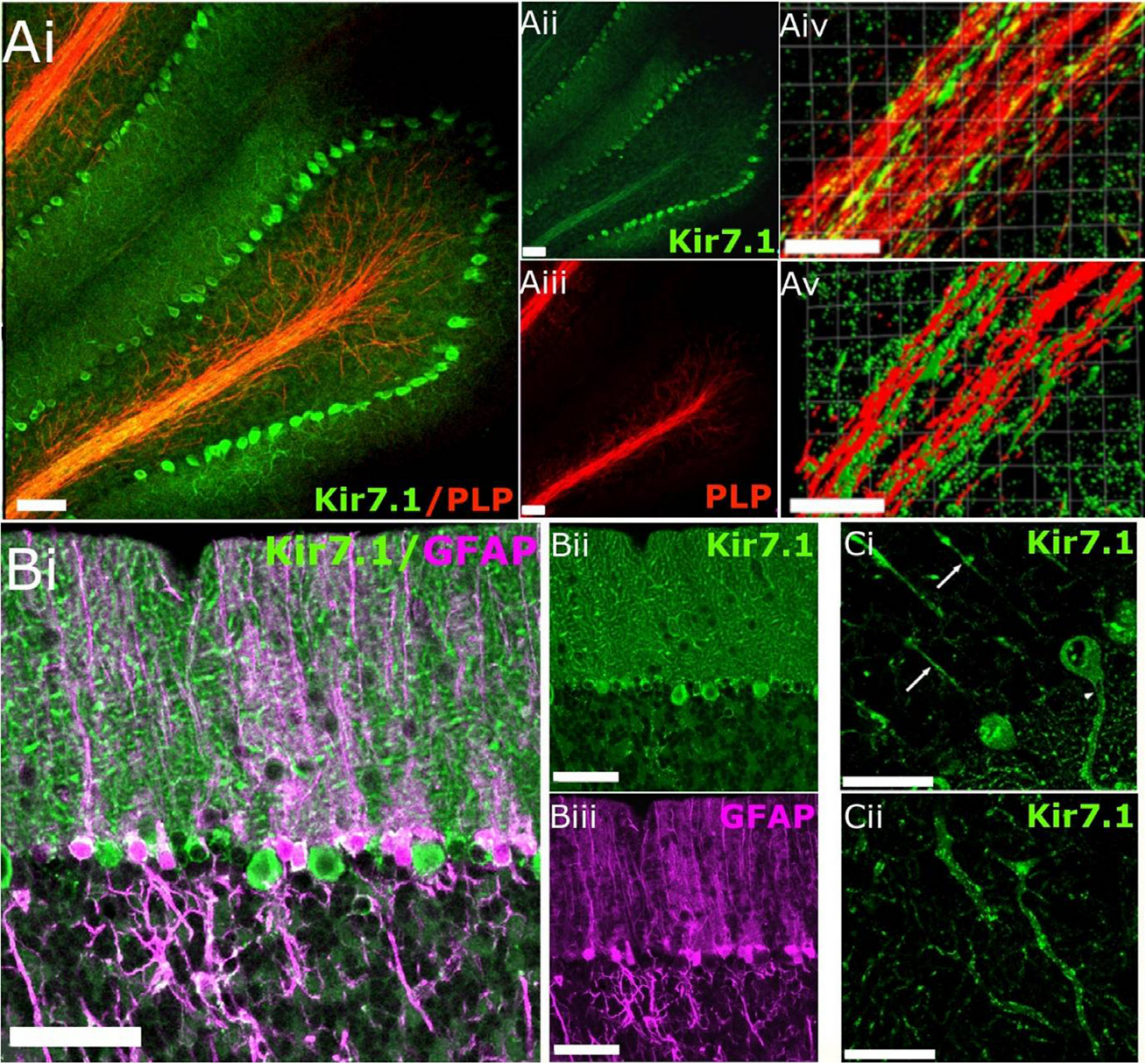
Kir7.1 immunostaining in the adult mouse cerebellum. (A) Immunostaining for Kir7.1 (green) in cerebellum from PLP‐DsRed mouse for myelin staining (red), where colocalisation appears yellow in the overlay (Ai); individual channels are illustrated for Kri7.1 (Aii) and PLP (Aiii). Higher magnification (Aiv) and isosurface imaging (Av) illustrate the apposition of Kir7.1 and PLP. (B) High magnification of the molecular layer (MCL) showing Kir7.1 (green) and GFAP (magenta), illustrating immunostaining of Bergmann glia cell bodies, where colocalisation appears white in the overlay (Bi); individual channels are illustrated for Kir7.1 (Bii) and GFAP (Biii). (Ci) Kir7.1‐immunopositive Purkinje cell somata, together with axons (Ci, arrows) and dendritic trees (Cii). Scale bars: 50 μm in all panels.

Immunohistochemical analysis of Kir7.1 expression in the brain is largely untested, with the exception that Kir7.1 has been shown to be strongly expressed in Purkinje neurones of the cerebellum (Krapivinsky et al. 1998) and the choroid plexus epithelium (Doring et al. 1998). Therefore, we first validated the anti‐Kir7.1 antibody by demonstrating intense immunostaining of Purkinje neurones (Fig. 1A) and the choroid plexus (Fig. 1B); there was a complete absence of immunostaining when sections were pre‐incubated with the blocking peptide against which the antibody was raised (Fig. 1A, inset). Skeletal muscle does not express Kir7.1, as demonstrated by Northern blot and rtPCR screening studies (Doring et al. 1998; Krapivinsky et al. 1998; Nakamura et al. 1999; Shimura et al. 2001), and the complete absence of immunostaining in skeletal muscle serves as a further negative control for the antibody (Fig. 1C). Furthermore, Western blot analysis of protein lysates from mouse cerebellum (CBL) and cortex (CTX) confirmed robust Kir7.1 protein expression with a predicted band at 54 kDa (Krapivinsky et al. 1998; Ango et al. 2004); positive bands were completely eliminated in the presence of the competitive peptide, which served as a further negative control (Fig. 1D). In some samples, very dim bands were observed at approximately 15 kDa, which corresponds to Kir7.1 protein that was proteolysed during sampling. As noted above, Purkinje neurones stood out in cerebellar sections immunolabelled for Kir7.1, but immunostaining was also evident along the myelinated fibre tracts, where it colocalised with PLP‐DsRed (Fig. 2A). In addition, double immunolabelling for Kir7.1 and GFAP indicated extensive colocalisation in the somata and processes of Bergmann glia (Fig. 2B, colocalisation appears white). Nonetheless, without doubt the most intense immunolabelling for Kir7.1 in the cerebellum was on Purkinje cell somata (Fig. 2Ai,ii, Bi,ii), together with their axons (Fig. 2Ci) and dendritic trees (Fig. 2Cii).

### Widespread neuronal and glial expression of Kir7.1 in the mouse forebrain

**Figure 3:**
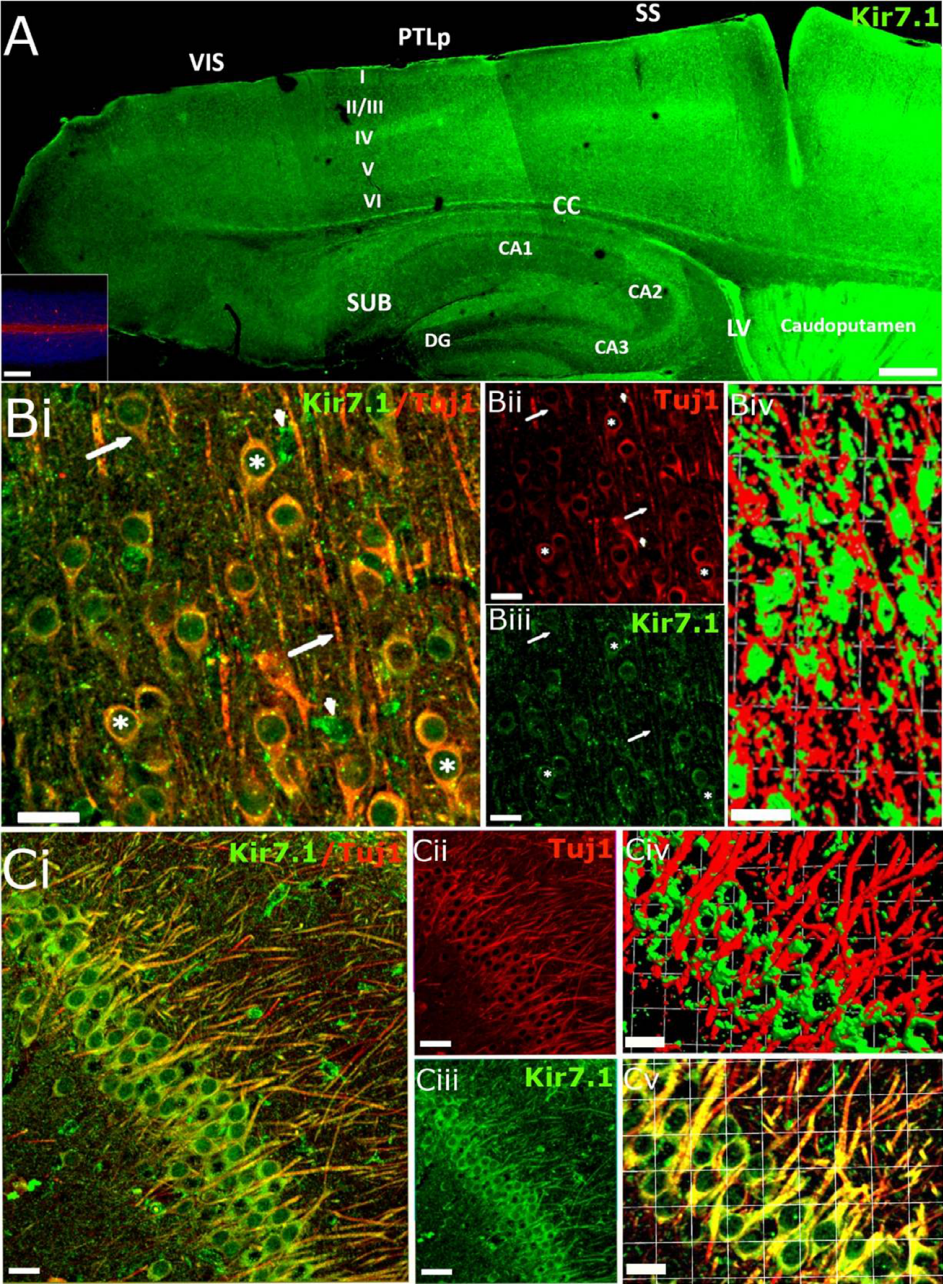
Kir7.1 immunostaining in the adult mouse forebrain. (A) Overview of the pattern of Kir7.1 expression in the adult mouse forebrain. Kir7.1 immunostaining was absent in negative controls pre‐incubated with peptide (A, Inset). (B,C) Double immunofluorescence labelling for Kir7.1 (green) and Tuj1 (red) in the cortex (B) and hippocampus (C). (B) Layer 2/3 cortical neurones expressing Kir7.1 on their cell bodies (asterisks) and axons (arrows); co‐expression appears yellow in the overlay (Bi) and Kir7.1 immunopositivity can be seen in Tuj1‐negative cells, which are likely to be astrocytes (arrowheads); individual channels are illustrated for Kir7.1 (Bii) and Tuj1 (Biii), together with isosurface images showing the close apposition of Kir7.1 and Tuj1 voxels (Biv). (C) Hippocampal pyramidal cells express Kir7.1 on cell somata and axons (Ci); individual channels are illustrated for Kir7.1 (Cii) and Tuj1 (Ciii), together with higher magnification of the overlay image (Cv) and isosurface image (Civ), illustrating the close apposition of Kir7.1 and Tuj1 voxels. Scale bars: (A) 300 μm; (A, Inset) 100 μm; (Bi‐iii and Ci‐iii)  25 μm; (Biv) 1 square unit = 35.42 μm; (Civ‐v) 1 square unit = 16.64 μm.

Kir7.1 immunostaining was also prominent in the cortex and hippocampus (Fig. 3A). Layer IV of the somatosensory cortex was strongly immunopositive for Kir7.1, together with the posterior parietal association area, extending all the way to the visual cortex (Fig. 3A). The most intense immunostaining for Kir7.1 was seen in the lining of the lateral ventricle (LV) and the caudate putamen, as well as the glia limitans delineating the body of the corpus callosum (Fig. 3A). In the hippocampus, areas CA1, CA2 and CA3 showed the greatest immunopositivity along with the molecular and polymorph layers of the dentate gyrus, while little immunostaining was evident in the dentate gyrus (Fig. 3A). Neuronal expression of Kir7.1 was confirmed using the neuronal marker Tuj1, as illustrated by the prominent immunostaining of somata, primary dendrites and axons in Layer II/III cortical pyramidal neurones (Fig. 3B) and hippocampal CA1 neurones (Fig. 3C).

**Figure 4:**
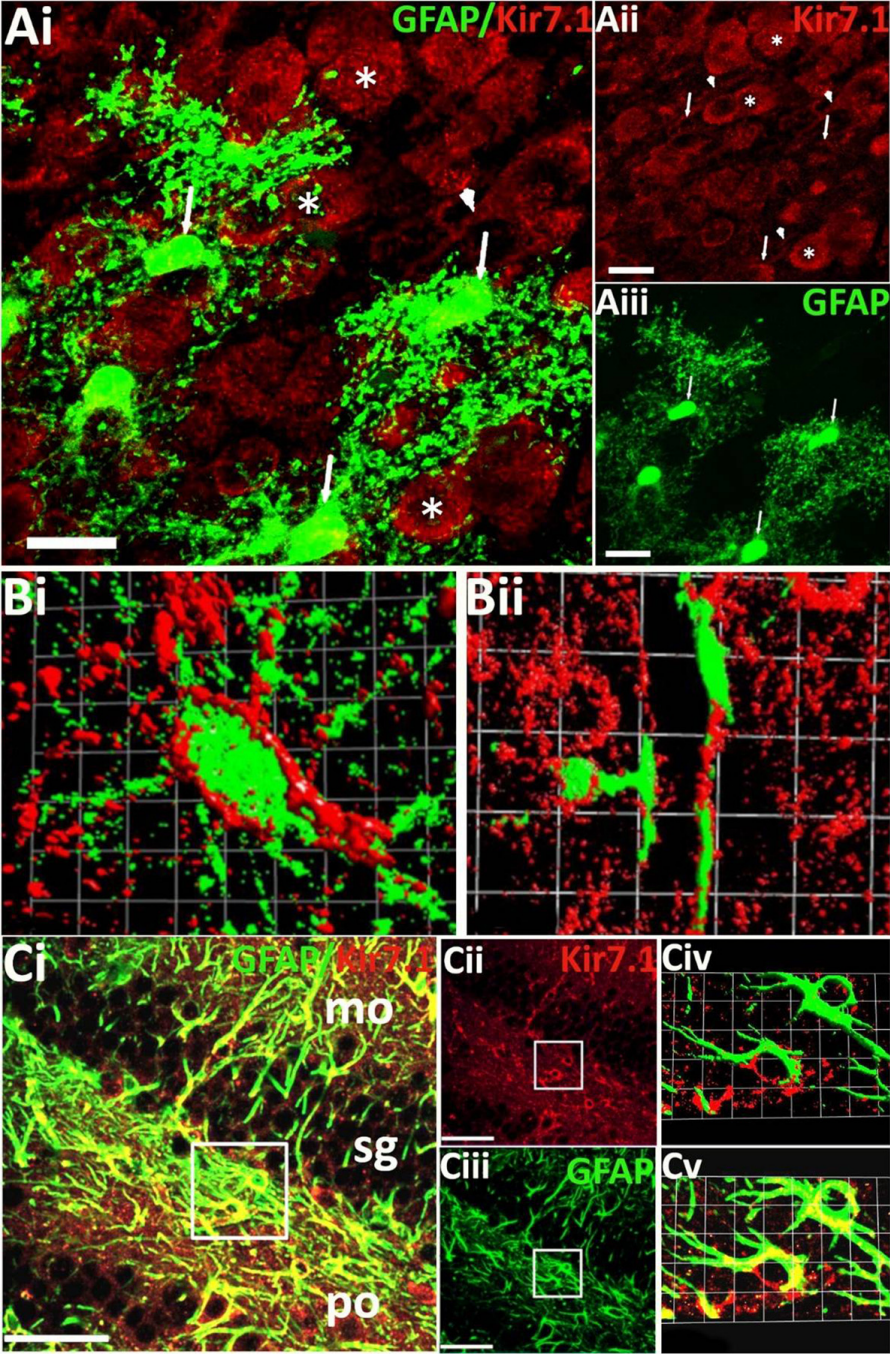
Astrocytic expression of Kir7.1. (A) Kir7.1 immunostaining (red) in cortical astrocytes (green) from adult GFAP‐EGFP mice, indicated by arrows in the overlay (Ai) and individual channels for Kir7.1 (Aii) and GFAP‐EGFP (Aiii). (B) High‐magnification 3D rendered isosurface images of a cortical Layer 1 astrocyte (Bi) and a perivascular astrocyte(Bii), showing the close apposition of Kir7.1 and EGFP voxels. (C) Double immunofluorescence labelling for Kir7.1 (red) and GFAP (green), illustrating Kir7.1‐immunopositive astrocytes in the hippocampus, where co‐localisation appears yellow in the overlay (Ci). Kir7.1 immunostaining is strongest in molecular (mo) and polymorph (po) layers, while the granule cell layer (sg) is less populated by astrocytes; individual channels are illustrated for Kir7.1 (Cii) and GFAP (Ciii), together with high‐magnification overlay (Cv) and isosurface image (Civ). Scale bars: (Ai‐iii) 30 μm; (Bi) 1 square unit = 5.47 μm; (Bii) 1 square unit = 25 μm; (Ci‐iii) 50 μm; (Civ‐v) 1 square unit = 6.06 μm.

Astroglial expression of Kir7.1 was examined in brain sections from GFAP‐EGFP mice, in which the astroglial gene glial fibrillary acidic protein (GFAP) drives expression of enhanced green fluorescent protein (EGFP), enabling astroglial three‐dimensional morphology to be visualised by confocal microscopy (Fig. 4). As illustrated in the frontal cortex, Kir7.1 immunostaining was evident in protoplasmic astrocytes, whose processes enwrapped Kir7.1 immunopositive neurones (Fig. 4Ai‐iii). Isosurface 3D rendering demonstrates Kir7.1 immunolabelling decorating GFAP‐EGFP+ astrocyte cell somata and processes (Fig. 4Bi), with high expression on perivascular end‐feet (Fig. 4Bii). Equivalent results were observed in hippocampal astrocytes, with evident colocalisation of Kir7.1 immunostaining with GFAP‐EGFP (Fig. 4C, colocalisation appears yellow).

**Figure 5:**
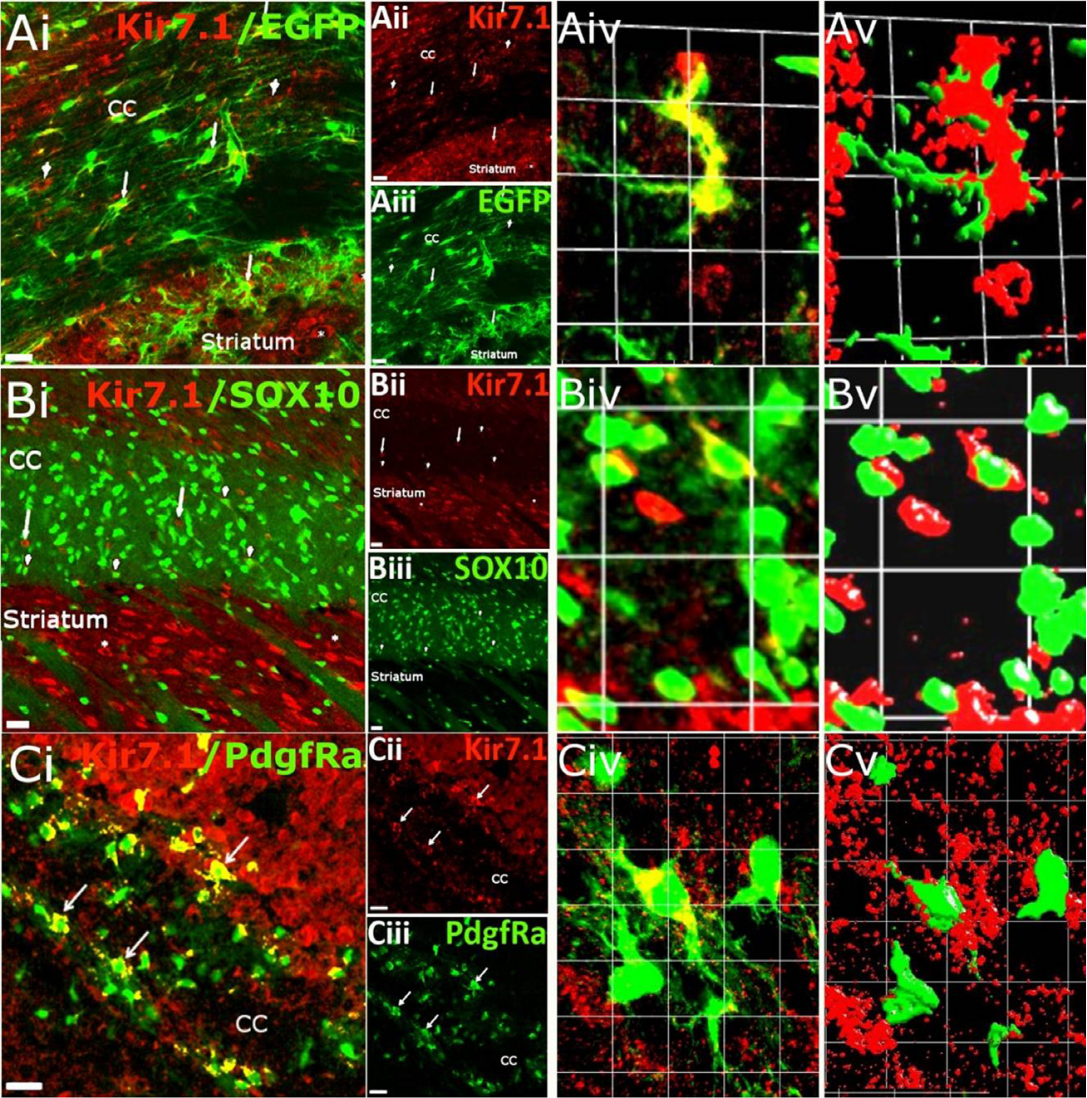
Kir7.1 expression in the corpus callosum (CC). (A) Kir7.1 immunostaining (red) in astrocytes (green) from adult GFAP‐EGFP mice, indicated by arrows in the overlay (Ai) and individual channels for Kir7.1 (Aii) and EGFP (Aiii); Kir7.1 immunostaining can also be seen in GFAP‐eGFP‐negative cells, which most likely are oligodendroglial cells (arrowheads) and striatal neurones (asterisks). High magnification of the overlay (Aiv) and isosurface image (Av) illustrates the close apposition of Kir7.1 and EGFP voxels (Av). (B) Sagittal section from an adult Sox10‐eGFP reporter mouse, in which some oligodendrocytes (green) are seen to be immunopositive for Kir7.1 (red), indicated by arrowheads (Bi), together with Sox10‐eGFP‐negative cells, which most likely are astrocytes (Bi, arrows), and the strongest immunopositivity in striatal neurones (Bi, asterisks); individual channels are illustrated for Kir7.1 (Bii) and SOX10 (Biii). High magnification of the overlay (Biv) and isosurface image (Bv) shows the close apposition of Kir7.1 and EGFP voxels. (C) Colocalisation of Kir7.1 immunostaining (red) in PdgfRa‐YFP positive OPCs (green, some indicated by arrows) in the corpus callosum of 15‐day‐old mice; colocalisation appears yellow in the overlay (Ci) and individual channels are illustrated for Kir7.1 (Cii) and PdgfRa‐YFP (Ciii). High magnification of the overlay (Civ) and isosurface image (Cv), showing the close apposition of Kir7.1 and PdgfRa‐YFP voxels. Scale bars: (Ai‐iii, Bi‐iii, Ci‐iii) 25 μm; (Aiv‐v) 1 square unit = 16.43 μm; (Biv‐v) 1 square unit = 37.36 μm; (Civ‐v) 1 square unit = 11.17 μm.

In the corpus callosum, the largest white matter tract in the brain, Kir7.1 immunolabelling was evident in both astrocytes and oligodendrocytes (Fig. 5). Colocalisation of Kir7.1 immunobalelling with GFAP‐EGFP was widespread in fibrous astrocytes of the corpus callosum and adjacent cortex and striatum (Fig. 5Ai‐iii). Astroglial expression of Kir7.1 on the cell somata and primary processes is clear in high magnification confocal images (Fig. 5Aiv) and corresponding isosurface rendered images (Fig. 5Av). Similarly, colocalisation of Kir7.1 immunolabelling in oligodendrocytes was identified by expression of EGFP driven by the oligodendroglial gene SOX10 (Fig. 5Bi‐iii); high magnification confocal images (Fig. 5B iv) and isosurface rendering (Fig. 5Bv) indicate oligodendroglial Kir7.1 immunostaining is heterogeneous and is localised to cell somata. To examine Kir7.1 in oligodendrocyte progenitor cells, we used Pdgfra‐creERT^2^‐Rosa26‐YFP mice (Rivers et al. 2008), which indicates most adult OPCs are immunopositive for Kir7.1 in the corpus callosum and surrounding grey matter (Fig. 5Ci‐iii). Higher magnification and isosurfacing rendering demonstrates Kir7.1 immunolabelling of OPC somata and fine processes (Fig. 5Civ‐v). The glial reporters (EGFP and YFP) are intracellular and the localisation of Kir7.1 at the cell surface of astrocytes (Fig. 5Av), oligodendrocytes (Fig. 5Bv) and OPCs (Fig. 5Cv) is consistent with Kir7.1 being functional plasmalemmal channels.

### Kir7.1 expression in the hindbrain

**Figure 6:**
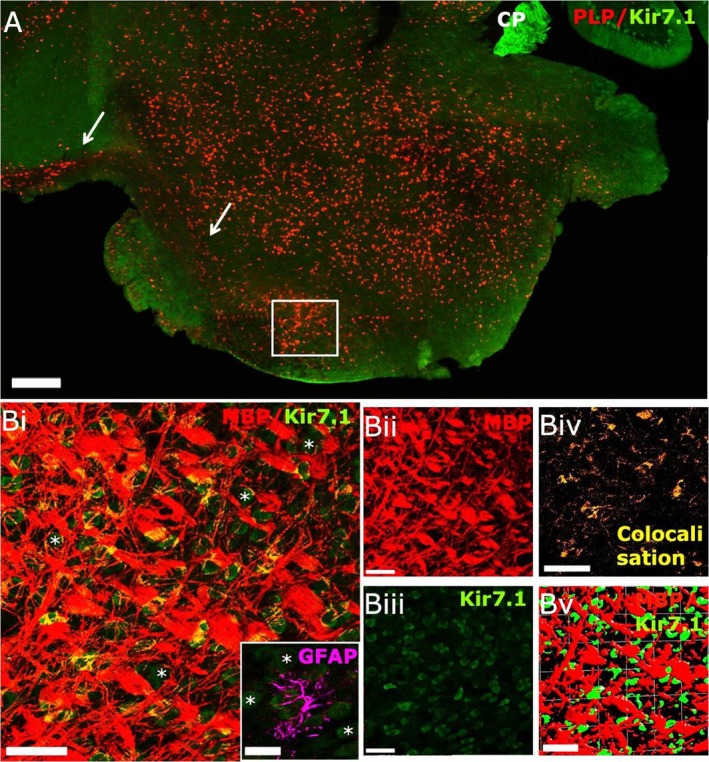
Kir7.1 expression in the mouse hindbrain. (A) Low‐magnification confocal image of sagittal section from adult PLP‐DsRed (red) reporter mouse immunolabelled for Kir7.1 (green). Kir7.1 is widely expressed in the pons, with intense expression in the choroid plexus of the third ventricle (CP), whereas the corticospinal tract appears immunonegative for Kir7.1 (arrows). (B) Higher magnification images from the area indicated by the white square in (A), immunostained for MBP (red) and Kir7.1 (green), showing neuronal expression of Kir7.1 (asterisks) and some co‐localisation of Kir7.1 and MBP(appears yellow in Bi); individual channels are illustrated for MBP (Bii) and Kir7.1 (Biii), together with the colocalisation channel (Biv) and isosurfacing image (Bv), which does not reveal very close apposition of Kir7.1 and MBP voxels (Bv), suggesting that Kir7.1 may be expressed by axons and not by myelin. (Bi‐inset) Double immunolabelling for Kir7.1 (green) and GFAP (magenta) did not detect expression of Kir7.1 in astrocytes of the pons. Scale bars: (A) 300 μm; (Bi‐v)  50 μm; (Bi, Inset) 25 μm.

Widespread immunolabelling for Kir7.1 was observed in the pons, except for the pyramidal tracts, which include the corticospinal and corticobulbar tracts (Fig. 6A, arrows). These largely Kir7.1‐immunonegative tracts contain the myelinated axons originating from the cerebral cortex, passing through the pons and terminating in the spinal cord and the brainstem, respectively. Higher magnification confocal images of sections double immunofluoresence labelled for Kir7.1 and the myelin marker MBP revealed a degree of co‐expression (Fig. 6Bi, iv, colocalisation appears yellow). However, the heaviest Kir7.1 immunostaining was in pontine neuronal somata (Fig. 6Bi,iii, some indicated by asterisks) and the isosurface rendering indicated Kir7.1 is expressed on axons and possibly not the surrounding myelin (Fig. 6Bv). Double immunolabelling for the astrocytic marker GFAP did not reveal Kir7.1 immunolabelling in astrocytes in the pons (Fig. 6A, Inset). Therefore, Kir7.1‐immunopositive neurones were prominent in the hindbrain, but glia appeared to be immunonegative for Kir7.1.

### Plasmalemmal expression of Kir7.1 in glia

**Figure 7:**
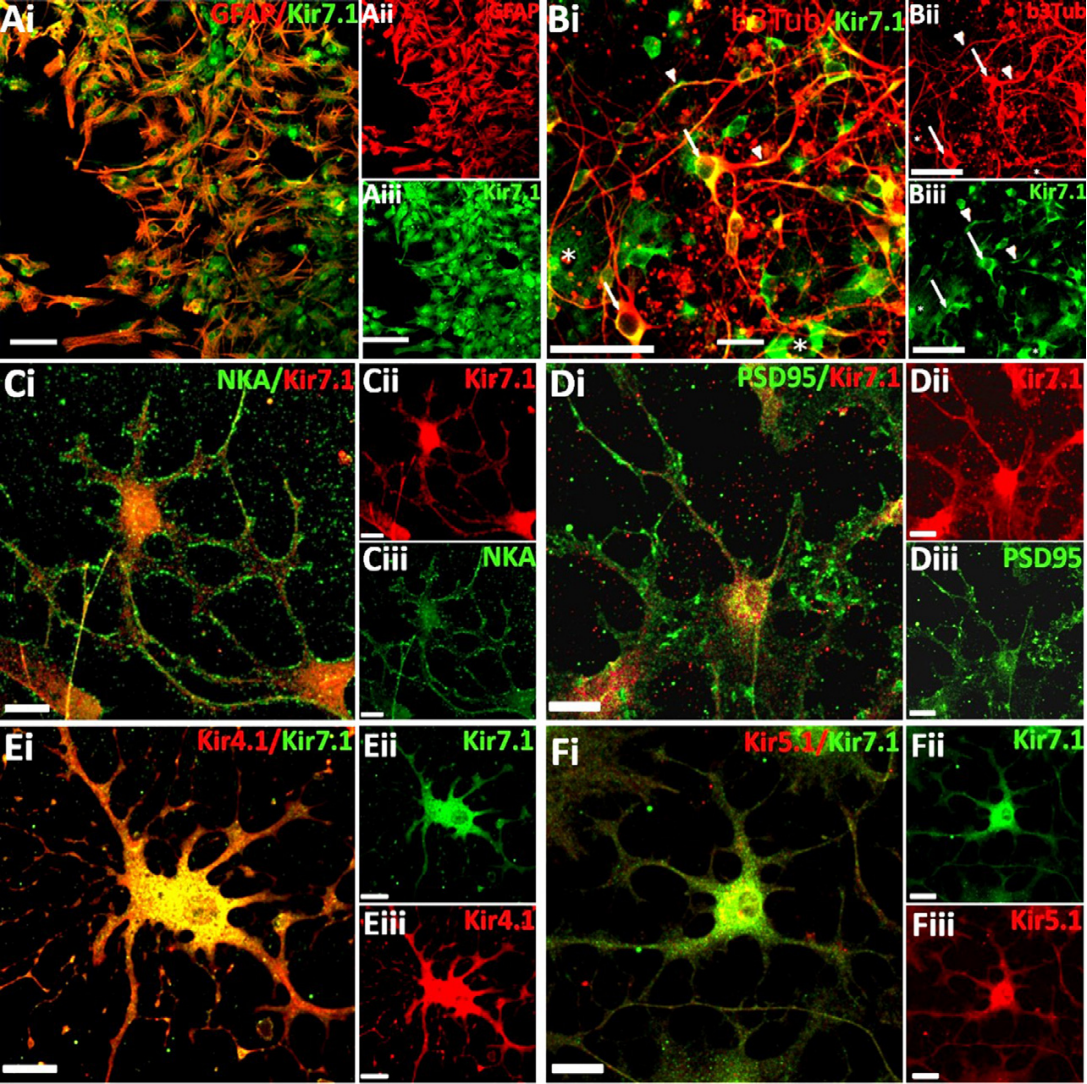
Neurons and glia express Kir7.1 *in vitro*. (A, B) Cells were isolated from P1‐2 mouse cortex and analysed after 14 days *in vitro* (DIV) by double immunofluorescence labelling for Kir7.1 (green) and the astrocyte marker GFAP (red, A) or neuronal marker Tuj1 (red, B, asterisks indicate Kir7.1+Tuj1‐ cells that are most likely astrocytes (C‐F) Optic nerve glial explant cultures from P7‐12 mice were analysed at 10DIV by double immunofluorescence labelling for Kir7.1 (green), with the plasmalemmal markers Na^+^/K^+^‐ATPase (C) and PSD95 (D), or the glial Kir channels Kir4.1 (E) and Kir5.1 (F), In all cases, overlays are illustrated (Ai, Bi, Ci, Di, Ei, Fi, co‐expression appears yellow), together with individual channels (Aii‐iii, Bii‐iii, Ci‐iii, Di‐iii, Ei‐iii, Fi‐iii). Scale Bars: A‐B = 50μm; C‐F = 20µm. [Colour figure can be viewed at wileyonlinelibrary.com]

The close apposition of glial cells and neurones can make it difficult to resolve unequivocal glial expression of Kir7.1 in brain sections. To test this, we performed Kir7.1 immunolabelling in cortical neurone‐astrocyte cultures and optic nerve explants cultures, neurones being absent from the latter (Fig. 7). Cortical cultures were prepared from P1‐2 mice and examined after DIV14; double immunofluorescent labelling demonstrates intense Kir7.1 immunostaining in GFAP‐immunopositive astrocytes (Fig. 7A) and Tuj1‐immunopositive neurones (Fig. 7B); colocalisation appears as yellow. For Kir7.1 channels to be functional in glia, the protein must be localised to the cell membrane. To test this, we performed double immunofluorescence labelling for Kir7.1 and plasmalemmal proteins in explant cultures prepared from optic nerves from P7‐P12 mice examined after DIV10. The results support co‐expression of Kir7.1 with plasmalemmal Na^+^/K^+^‐ATPase (Fig. 7C) and PSD95 (Fig. 7D), together with the main glial channel Kir4.1 (Fig. 7E) and Kir5.1 (Fig. 7F).

## Discussion

Kir7.1 has not been extensively studied in the CNS. Here, we show that Kir7.1 immunolabelling in the brain is more widespread than previously assumed and demonstrate Kir7.1 are not solely neuronal channels but are also expressed by glial cells, albeit heterogeneously. In neurones, Kir7.1 regulate excitability and our data indicate this function may be widespread in the brain. In addition, Kir7.1 are known as K^+^ transporting channels and their expression in glial cells indicates they play an important role in the primary glial function of K^+^ homeostasis.

In the absence of brain tissue from Kir7.1 knock‐out mouse, which do not survive postnatally (Villanueva et al. 2015), we used skeletal muscle and cerebellum as negative and positive controls for the immunostaining observed in our study. We demonstrate the complete absence of immunostaining in skeletal muscle, which does not express Kir7.1 (Doring et al. 1998; Krapivinsky et al. 1998; Nakamura et al. 1999; Shimura et al. 2001). In contrast, cerebellar Purkinje neurones exhibited the heaviest immunostaining for Kir7.1, which is fully corroborated by the findings of Krapivinsky et al. (1998). Overall, our results provide evidence of Kir7.1 immunostaining in glial cells and indicate that Kir7.1 may be more widely distributed in neurones than previously supposed.

In neurones, Kir7.1 are localised to cell somata, primary dendrites and axons, consistent with a role for Kir7.1 in the summation of electrical signals received by these neurones and axon potential propagation. Kir channels help maintain the dendritic resting membrane potential (RMP) and hence regulate postsynaptic excitability (John & Manchanda, 2011). Moreover, Kir7.1 activity is modulated by activation of G‐protein coupled receptors (GPCRs) and cAMP, for example beta‐2 adrenergic receptor (β2AR) and MC4R (Ghamari‐Langroudi et al. 2015; Carrington et al. 2018), whereas increased intracellular cAMP increases Kir7.1 (Zhang et al. 2008). Notably, Kir7.1 are most widely and prominently expressed in neuronal postsynaptic membranes, equivalent to GIRK/Kir3.x (Inanobe et al. 1999; Koyrakh et al. 2005), which have been shown to be intimately involved in the modulation of neuronal excitability in CA1 hippocampal neurones (Koyrakh et al. 2005). In addition, we observed heterogeneous expression of Kir7.1 in the pons, which encompasses a number of grey matter nuclei associated with aspects of respiratory control and sleep, as well as sensory and motor relays to and from cranial nerves. A complete analysis of the specific pontine neuronal types expressing Kir7.1 was beyond the scope of the current study, but Kir4.1 and Kir5.1 have been shown to be important in central chemoreception (D'Adamo et al. 2011) and it will be of interest to determine whether Kir7.1 are also involved. Significantly, an important role for Kir7.1 in neurones is indicated by the neurological phenotypes observed with KCNJ13 mutations (Pattnaik et al. 2015), in particular the genetic disease Leber's congenital amaurosis, where variable degrees of cerebral, neurological and neurodevelopmental (mental retardation) abnormalities have been reported, including cerebellar malformation and severe motor deficits (Weinstein et al. 1984; Fazzi et al. 2005; Petraglia et al. 2012). Our results support the need for further functional and expression studies to determine the function of Kir7.1 in regulating neuronal activity.

The homeostatic functions of glia are critical for brain function (Verkhratsky & Nedergaard, 2018) and diverse Kir channels are implicated in these functions (Butt & Kalsi, 2006). Ours is the first study indicating that Kir7.1 may also be important in astrocytes and oligodendrocytes. Numerous studies have demonstrated the importance of Kir4.1 in maintaining the strongly negative resting membrane potential of glia and providing the molecular basis for glial K^+^ uptake (Orkand et al. 1966; Newman et al. 1984). However, synaptic transmission and normal rhythmic activity in the hippocampus are not affected in Kir4.1 knock‐out mice (Neusch et al. 2006; Djukic et al. 2007). This suggests that other Kir channels are important in K^+^ homeostasis, such as Kir7.1, which is less sensitive to Ba^2+^ blockade. Notably, K^+^ in the rat hippocampus and optic nerve are dependent on glial and axonal Na^+^/K^+^‐ATPase and not Ba^2+^‐sensitive Kir4.1 channels (Ransom et al. 2000; D'Ambrosio et al. 2002; Meeks & Mennerick, 2007). It is significant, therefore, that in the transporting epithelia of the choroid plexus, kidney tubules, RPE and thyroid follicular cells, Kir7.1 channels colocalise with Na^+^‐K^+^‐ATPase and have a “K^+^ recycling’ function, providing K^+^ efflux to balance influx of K^+^ through Na^+^‐K^+^ pumps (Ookata et al. 2000; Shimura et al. 2001; Yang et al. 2003; Wimmers et al., 2007). Our co‐localisation *in vitro* results support an equivalent role for Kir7.1 and Na^+^‐K^+^‐ATPase in glia.

Astrocytes also play a critical role in CNS water homeostasis, which is a key function of Kir7.1 in transporting epithelis. Astrocytes contacting blood vessels, ventricles and the pia show high expression of aquaporin 4 (AQP4) which colocalises with Kir4.1 (Nielsen et al. 1997). An equivalent water homeostatic function is performed by the RPE and renal epithelial cells and is dependent on Kir7.1 (Wimmers et al. 2007; Hejtmancik et al. 2008). Our results support the possibility that astroglial Kir7.1 have an equivalent function, as it is robustly expressed on perivascular endfeet. The question remains as to the possible function of Kir7.1 in OPCs and oligodendrocytes, for which expression was more heterogeneous than in astrocytes. The function of oligodendrocytes is myelination, and so it is reasonable to assume that Kir7.1 are involved in this function, as has been shown for Kir4.1 (Neusch et al. 2001). OPC maturation into oligodendrocytes and myelination are dependent on the development of a strongly negative RMP, which is related to a loss of Kv and upregulation of Kir (Knutson et al. 1997; Neusch et al. 2003). Moreover, oligodendrocytes and OPCs are exposed to large shifts in ions and water during axonal action potential propagation (Berger et al. 1991; Kettenmann et al. 1991; Menichella et al. 2006). It is likely that Kir7.1 play an important role in ion and water homeostasis in oligodendrocytes.

In summary, the key finding of this study is that Kir7.1 immunolabelling is widespread in the adult mouse brain. Our results provide new information on the pattern of Kir7.1 expression in neurones and glia. At present, the physiological function of Kir7.1 in neurones and glia is unresolved, but Kir7.1 are likely to play important roles in regulating neuronal excitability and glial K^+^ and water homeostasis.

## Conflict of interest

A.B. is a shareholder and co‐founder of GliaGenesis Ltd.

## Author contributions

Maria Papanikolaou: data acquisition‐analysis/interpretation, drafting of the manuscript. A.L. and A.B.: data analysis/interpretation, critical revision of the manuscript.
